# Predation Risk, Resource Quality, and Reef Structural Complexity Shape Territoriality in a Coral Reef Herbivore

**DOI:** 10.1371/journal.pone.0118764

**Published:** 2015-02-25

**Authors:** Laura B. Catano, Bridgette K. Gunn, Megan C. Kelley, Deron E. Burkepile

**Affiliations:** Marine Science Program, Department of Biological Sciences, Florida International University, North Miami, Florida, United States of America; The Australian National University, AUSTRALIA

## Abstract

For many species securing territories is important for feeding and reproduction. Factors such as competition, habitat availability, and male characteristics can influence an individual’s ability to establish and maintain a territory. The risk of predation can have an important influence on feeding and reproduction; however, few have studied its effect on territoriality. We investigated territoriality in a haremic, polygynous species of coral reef herbivore, *Sparisoma aurofrenatum* (redband parrotfish), across eight reefs in the Florida Keys National Marine Sanctuary that were either protected or unprotected from fishing of piscivorous fishes. We examined how territory size and quality varied with reef protection status, competition, predation risk, and male size. We then determined how territory size and quality influenced harem size and female size to understand the effect of territoriality on reproductive potential. We found that protected reefs trended towards having more large predatory fishes and that territories there were smaller but had greater algal nutritional quality relative to unprotected reefs. Our data suggest that even though males in protected sites have smaller territories, which support fewer females, they may improve their reproductive potential by choosing nutritionally rich areas, which support larger females. Thus, reef protection appears to shape the trade-off that herbivorous fishes make between territory size and quality. Furthermore, we provide evidence that males in unprotected sites, which are generally less complex than protected sites, choose territories with higher structural complexity, suggesting the importance of this type of habitat for feeding and reproduction in *S. aurofrenatum*. Our work argues that the loss of corals and the resulting decline in structural complexity, as well as management efforts to protect reefs, could alter the territory dynamics and reproductive potential of important herbivorous fish species.

## Introduction

Territories often serve as both feeding and breeding grounds that provide nutritional and reproductive benefits for male territory holders [1]. For haremic territorial species, territory size can influence a male’s ability to attract and mate with females, ultimately affecting his reproductive success [2]. Multiple factors can influence the size of territories including the density of competitors, traits of the territory holder such as body size, and predation risk. At high competitor densities, for example, territory holders must increase the time and energy spent defending borders and evicting intruders, often resulting in decreased territory size [3,4] except for the largest, competitively superior males [5]. Although a number of studies have investigated the influence of competition and male traits on territory dynamics, fewer have focused on the effects of predation risk, which is predicted to be an important cost of defending territories [6]. Given the strong influence of predation risk on influencing foraging behavior [7–9], one would expect similar impacts on territoriality. Large and/or high quality territories can enhance breeding success [3,10–12] and thus can have a strong effect on regulating population densities [13–15]. Therefore, to understand the population dynamics of territorial species it is essential to know the factors that influence territoriality.

On coral reefs, many groups of fishes such as Pomacentridae [16], Chaetodontidae [17], and Labridae [18] exhibit conspicuous territorial behavior. For those in the family Scaridae, tribe Scarini, known as parrotfishes, a group of harem females occupy the territory of a terminal phase male and breed with him [19–22]. Territoriality in this group provides both nutritional [23] and reproductive benefits [19,24]. For instance, by defending territories against individuals with the highest resource overlap, particularly conspecific males, territory holders gain exclusive access to food resources and spawning privileges with harem females within their territories [19,22]. One of the primary fitness costs of territoriality is aggressive defense against competitors, which decreases time available for foraging and mating. Thus, where competitors are abundant, territories are generally smaller [24].

An often overlooked cost of territoriality for parrotfishes is a potential increase in vulnerability to predation [25]. Frequent and active defense of large territories against intruders may put territory holders at a greater risk of predation. Mating behaviors may also increase predation risk [26], resulting in a trade-off between mating behaviors and anti-predator behaviors when predators are abundant [27]. Indeed, evidence from multiple systems suggests that increasing predation risk alters mate choice [28,29], male mating tactics [30], the timing of mating [31], and courtship [32] (for review see: [33]). However, relatively few studies have investigated the effect of predation risk on territoriality in fishes [5,34,35], despite its importance in mating success for many species. Given that the abundance of large predators will vary greatly depending on if coral reefs are protected or vulnerable to fishing pressure, it is important to consider how variable levels of predation risk impact the territoriality and reproductive success of parrotfishes. Large grouper, sharks, and barracuda are increasing in size and abundance inside many protected areas [36] which will likely increase the vulnerability of non-targeted species (i.e., parrotfishes) to predation risk and potentially alter their territorial and mating behaviors.

Territory selection and defense is a complex process that forces males to balance potentially conflicting forces of territory size, diet quality, structural complexity, competition and predation. For instance, increasing territory size may not improve resource availability for territory holders, particularly if the cost of aggressive defense is high, because resources are patchily distributed on reefs [37] and vary in nutritional quality [38]. Furthermore, the associated costs and benefits of territory selection may require that organisms make trade-offs. For example, choosing a territory with high structural complexity may provide benefits including refuges from predation and greater resource diversity [39]. However, this may come at a cost of higher competition because complex habitats likely attract increased densities of competitors [40]. Thus, understanding the drivers of territoriality requires using multiple metrics of territory quality to assess the costs and benefits of holding territories. However, many studies focus only on a limited subset of potential drivers, which may underestimate the potential tradeoffs of different mechanisms influencing territoriality.

In this study we investigated the causes and consequences of territoriality in the herbivorous parrotfish, *Sparisoma aurofrenatum*, on coral reefs in the Florida Keys National Marine Sanctuary (FKNMS). *Sparisoma aurofrenatum*, an abundant herbivore across Caribbean reefs [36,41], are protogynous with three distinct color phases; juvenile phase, initial phase and terminal phase. Terminal phase (TP) individuals are males which usually maintain permanent territories and spawn year-round with their harem of females [21]. Spawning generally occurs daily during mid- to late-afternoon [21,42]. It is rare that TP males are seen together, except when involved in aggressive interactions along the borders of their territories [19,43]. Initial phase (IP) individuals may be either females or males that have not yet transformed to TP male morphology. Female IP *S. aurofrenatum* are generally either solitary or move in small groups within a TP male’s territory (pers. obs.). The diet of *S. aurofrenatum* consists of primarily macroalgae and algal turfs [44,45].

We measured various characteristics of *S. aurofrenatum* territories and harems to test if: (1) reef protection status, competitor and predator biomass, and male size influenced territory size and quality, (2) territory size, territory quality, and male size influenced the size and number of harem females, and (3) territory and harem characteristics influenced the frequency of aggressive and reproductive interactions. We used multiple metrics to characterize territory quality including algal abundance, algal nutritional quality, and reef structural complexity. We expected territory size to decrease with increasing predator and competitor biomass because of the increased cost of territoriality. We also expected that larger males, which are likely competitively superior and at less risk of predation, would have larger territories. Further, we anticipated that territory size and territory quality would be inversely proportional and that large and/or high quality territories would be associated with large females and harems. Finally, we predicted that increasing predation risk would reduce the frequency of aggressive and reproductive interactions but that these interactions would be positively associated with harem size and the quality and size of territories.

## Materials and Methods

### Ethics Statement

This work was conducted with permission from the Florida Keys National Marine Sanctuary under permit no. FKNMS-2012–080 and the protocol for this study was approved by The Florida International University Institutional Animal Care and Use Committee (IACUC), (Protocol Approval #12–015, FIU Animal Welfare Assurance Number #A3096–01).

### Site Description

The Florida Keys reef tract is a large bank reef system located approximately 8 km offshore of the Florida Keys, USA, parallel to the island chain. Carnivorous fishes (e.g. snapper, grouper, barracuda) are heavily exploited in the region by both commercial and recreational fisheries [46,47]. Fishing activity is restricted inside 23 no-take zones, which were established in 1997 within the Florida Keys National Marine Sanctuary (FKNMS) [48]. Piscivorous fishes including black grouper (*Mycteroperca bonaci*) and mutton snapper (*Lutjanus analis*) have increased in size and abundance within these protected areas [36,48]. Unlike most piscivores, herbivorous fishes (e.g., parrotfish, surgeonfish) are not heavily targeted by fishing across the entirety of the FKNMS (i.e., in both protected and unprotected zones). Although some regulated take of herbivores as ornamental fishes is allowed, their populations are robust in the FKNMS relative to most other reefs in the wider Caribbean [49]. Voluntary compliance with sanctuary regulations restricting fishing is reported to be high based on opinion polls of boat users in the FKNMS, even though the sanctuary relies heavily on interpretive enforcement (i.e., enforcement primarily through education) [50]. The FKNMS is an ideal region to test hypotheses about the functional impact of predators on herbivorous fish because it does not confound predator effects (e.g. protected vs. unprotected areas) with vast differences in herbivore abundance across reefs since herbivores are protected everywhere.

**Fig 1:**
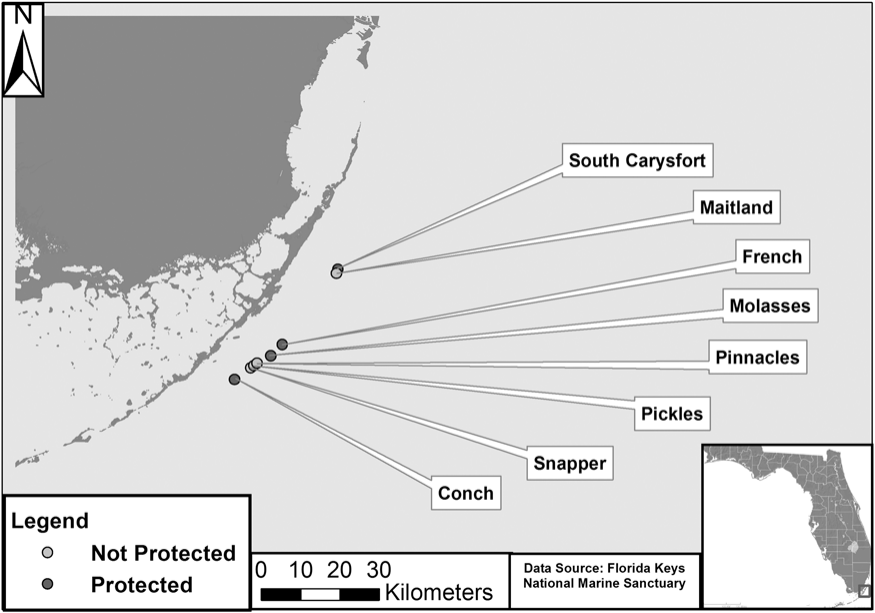
Study sites. Map of study sites sampled in the northern reef tract of the Florida Keys National Marine Sanctuary.

We sampled four protected (South Carysfort, Molasses, French, and Conch) and four unprotected (Pickles, Pinnacles, Maitland, and Snapper Ledge) forereef sites along the northern reef tract off of Key Largo (Fig. 1, S1 Table). Sites were similar in depth (6–8 m) and physical parameters (e.g., rugosity) and were separated by at least 700 m to assure independence. It is unlikely that most reef fishes, with the exception of large, mobile predators such as jacks, would move among reefs over such distances [51], particularly when separated by open areas (i.e., large expanses of sand or rubble) [52], as was the case with the sites used in this study. Focal fish observations were made between June-July 2012 on the forereef at depths of 6–8 m.

### Territory Delineation and Behavioral Observations

At each site we delineated the territories of 20 TP males on SCUBA, using a towed surface float that was attached to a handheld GPS (Garmin eTrex 10, accurate to < 3 m; see [53,54] for similar methods). Divers located a TP male and maintained a position at least 1 m behind and 1 m above the focal fish during a 25-min behavioral follow while towing the surface float with GPS. Data collection began after 5-min to allow fish to acclimate to diver presence. We maintained the 1 m distance from focal males to limit diver influence on their behavior. Males generally acclimated well to diver presence, likely because *S. aurofrenatum* are not targeted by spear-fishing within the FKNMS and do not perceive divers as a threat [55]. However, if males altered their activity in response to our presence (e.g., hiding or swimming rapidly away from the diver), we immediately stopped the observation and excluded these individuals from further study and analysis. Based on longer, 30-min observations, we determined that males patrolled the full extent of their territories several times in the first 20-min of each observation (S1 Fig.). Therefore, we limited all data acquisition to 20-min periods. We geo-referenced tracks from the GPS units using ESRI ArcGIS version 10.0 (Redlands, CA). We determined territory sizes by calculating minimum convex polygons of geo-referenced points for the total area covered from the entire observation. At each site, observations were performed over the same time period (10:00–16:00 h) because there are significant diurnal changes in activity for many parrotfishes [56]. Sites were sampled only on clear days with calm seas (< 0.75 m) to obtain the most accurate GPS signals.

During the 20-min periods of data collection, we recorded aggressive interactions and spawning activities. Aggressive interactions included jaw fighting, parallel swimming, pectoral fin displays, and rapid chasing that was initiated by or was directed towards the focal male. Spawning events are conspicuous and involve the focal male and a female swimming alongside each other and ultimately rushing towards the surface and releasing gametes into the water. We also recorded other focal male reproductive interactions with harem females that did not terminate with spawning [i.e., “looping”, a down and up movement that males performs near females to initiate courtship [42,57]]. We synchronized the watch of each diver with the GPS unit and recorded the time of behavioral observations. The geo-referenced tracks then allowed us to determine the exact position where each behavior occurred within the territory.

### Territory Metrics

We estimated harem size by recording the number of females permitted within the territory by the focal male during the 20-min territory survey. Immediately after the survey, divers haphazardly swam the extent of each territory and counted females to verify counts made during the observation and to ensure that females were not counted more than once. Females tended to loosely aggregate in groups within territories, facilitating accurate counts of harem size. We designated individuals to be harem females if they exhibited IP coloring and were not chased by the TP male because TP males often chase both IP males and non-harem females from their territories [21]. The fork length of the focal TP male and the females within his territory were estimated visually. Prior to data collection, the observers were trained to estimate fish size by assessing the length of static objects underwater (i.e., sections of PVC pipe cut to various lengths) until they could reliably estimate length to the nearest 1 cm. Accuracy was confirmed approximately every two weeks using this same methodology.

After the 20-min focal follow, we assessed benthic community composition and collected samples for algal nutritional quality within each territory. We collected these metrics along four 5 m transects radiating from the territory center point in the north, south, east and west directions. We standardized transects to this length based on the average territory diameter (~ 10 m). We verified in situ estimations of territory centers with geo-referenced points. Along each transect, photographs were taken every meter to produce twenty 50 cm x 50 cm photo-quadrats located on the benthos. To quantify benthic cover, 25 points were overlaid on these photographs in a 5x5 grid and analyzed for cover of benthic organisms using Coral Point Count V4.1 [58] to produce a total of 500 points per territory. Categories were created for: (1) crustose coralline algae, short algal turf (algal filaments < 0.5 cm tall) and bare space (abbreviated CTB—‘crustose, turf, bare’), (2) turf algae (algal filaments > 0.5 cm tall) and sediment (abbreviated TAS—‘turf algae, sediment’), (3) sponges, (4) gorgonians and (5) zoanthids. Macroalgae were classified to genus and scleractinian corals to species. Along each transect we collected portions (n = 4/territory) of *Dictyota menstrualis*, a commonly consumed species of macroalgae [45], to analyze carbon and nitrogen content (a metric of resource quality). After collection, samples were immediately placed on ice and later transported back to the lab where they were kept frozen until dried at 60°C. To obtain average C and N measurements for each territory, the four samples from each territory were combined and ground to a fine powder with a mortar and pestle, then weighed and processed using a CHN elemental analyzer.

We determined physical habitat characteristics including depth and rugosity (i.e., structural complexity) of each territory using a LiDAR (Light Detection and Ranging)-derived bathymetric data set provided by the U.S. Geological Survey (USGS) (available online http://pubs.usgs.gov/of/2007/1395/start.html). We used this dataset to create a raster of benthic rugosity (1 m x 1 m resolution) with the Benthic Terrain Modeler (a collection of ESRI ArcGIS-based tools available online http://www.csc.noaa.gov/digitalcoast/tools/btm/index.html). Using the ArcGIS zonal statistics tool we calculated average depth and rugosity within each territory’s minimum convex polygon. LiDAR-derived rugosity measurements are often significantly positively correlated with traditional transect estimates of rugosity [59–61]. Furthermore, by using LiDAR data we obtained fine-grain (1m^2^) metrics of rugosity that covered large extents (i.e., the entirety of each male’s territory). Thus, we are likely capturing the grain and extent at which *S. aurofrenatum* makes foraging, sheltering and reproductive decisions—all of which are potentially important to consider when evaluating territory dynamics. Rugosity on such spatial scale would have been logistically impossible using traditional metrics.

### Site Characteristics

At each site, we also estimated benthic cover, algal nutritional quality, and rugosity outside of parrotfish territories so that we could compare them to those metrics measured inside territories. By doing so, we obtained reference metrics to determine if TP males selected territories with certain characteristics that differed from the surrounding reef. To obtain site-wide estimates of benthic cover and algal nutritional quality, we conducted eight 25 m transect surveys that were haphazardly laid out parallel to the main reef formation. We took photo-quadrats every meter and collected portions of *D. menstrualis* along each transect following the methodology described above for collection and processing of these data. We pooled the benthic data points from all eight transects and randomly resampled 500 points from the pooled data to calculate reference percent cover metrics. Repeating this procedure twenty times allowed us to obtain site estimates of benthic cover that were comparable to and estimated with the same precision as those inside territories (i.e., based on 500 benthic data points). Performing this bootstrapping procedure was necessary because there was a limited area to conduct transects in surrounding reef without encountering *S. aurofrenatum* territories on smaller reefs. Finally, to obtain site-wide estimates of rugosity comparable to the 20 territory estimates, we haphazardly placed twenty 100 m^2^ plots (the average size of the TP male territory) using ArcGIS that did not overlap with our measured *S. aurofrenatum* territories. We then calculated rugosity within plots based on LiDAR data using the ArcGIS zonal statistics tool. We used benthic habitat maps available from the FKNMS (http://flkeysbenthicmaps.noaa.gov/) to distinguish reef from non-reef habitats (e.g., seagrass, sand, rubble).

To examine potential relationships between territory/harem size and competitor or predator abundance, we used fish abundance and biomass estimates from surveys done at each site. These data were collected from eight 25-m long transects along which we identified and visually estimated the fork length of all fishes within a 4-m wide window. We used published length:weight relationships to convert fish lengths to biomass [62].

### Statistical Analysis

We first tested the hypothesis that protection status, competitor and predator biomass, and focal male length influenced territory metrics (i.e., territory size, rugosity, algal nutritional quality and algae percent cover). To test for differences in competitor biomass, predator biomass, and territory sizes between protected and unprotected sites we used Welch Two-Sample t-tests. We used two-way Analysis of Variance (ANOVA) followed by Tukey HSD post-hoc tests to examine the effect of protection status and territory status (i.e., reference vs. territory) to test if differences in territory quality variables (e.g., algal abundance and algal nutritional quality) were due to protection status or due to the attributes of the reefs themselves. We used Analysis of Covariance (ANCOVA) to examine the effect of male size on territory quality variables among protected and unprotected sites.

We investigated the effect of site-level variables including competitor and predator biomass on territory quality metrics using mixed-effects models with site and status modeled as random effects. We defined competitor biomass as the combined biomass of *Sparisoma* species because the overwhelming majority of aggressive interactions were with conspecifics and congeners (see Results). We estimated predator biomass, which in our prior work was shown to be a useful metric to estimate predation risk [45], by summing all primarily piscivorous fishes of the families Carangidae, Lutjanidae, Serranidae and Sphyraenidae that were > 30 cm and known to consume adult parrotfishes [based on [44]]. We included only piscivorous Lutjanid species that likely represented the greatest threat to *S. aurofrenatum*, including: *Lutjanus analis*, *Lutjanus apodus*, *Lutjanus jocu*. Other Lutjanid species (e.g., *Lutjanus griseus*, *Lutjanus synagris*, *Lutjanus mahogoni*), which tend to target small crustaceans and decapods [44,63,64], would be unlikely to threaten an adult *S. aurofrenatum*, and thus were not included in the predator biomass metric. Because larger males could have a differential ability to procure higher quality territories, we examined the effect of male size on territory quality metrics using simple linear regressions. We also tested if male size, female size, and average harem size were different between protected and unprotected sites using Welch Two-Sample t-tests. Next, we tested the hypothesis that territory size, territory quality, and male size influenced the size and number of harem females. We used multiple linear regressions to examine how territory metrics (i.e., size, rugosity, algal nutritional quality and algal percent cover) and male length influenced both harem size and average female size.

Finally, we tested the hypothesis that territory and harem characteristics would influence the frequency of aggressive and reproductive interactions. We used multiple logistic regression to test the effects of these variables on the probability of occurrence of spawning and aggressive behaviors. To understand which fish species were the major targets of aggression, we tallied the aggressive interactions by species for each male and used a one-way ANOVA followed by Tukey HSD post-hoc tests to make comparisons. To understand how spawning events varied with protection status, we used a chi-square analysis to compare the proportion of males spawning in protected and unprotected areas. All statistical analyses were conducted using R version 3.0.1. Parametric assumptions of normality and homoscedasticity were verified using plots of the residuals.

## Results

**Fig 2:**
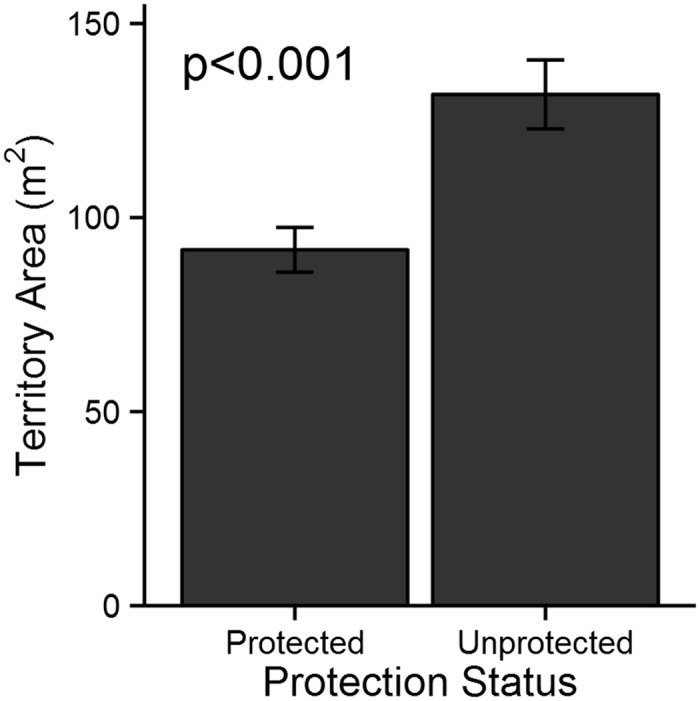
Territory sizes. Mean (± SE) of territory sizes (m^2^) in protected (n = 77) and unprotected areas (n = 79).

**Fig 3:**
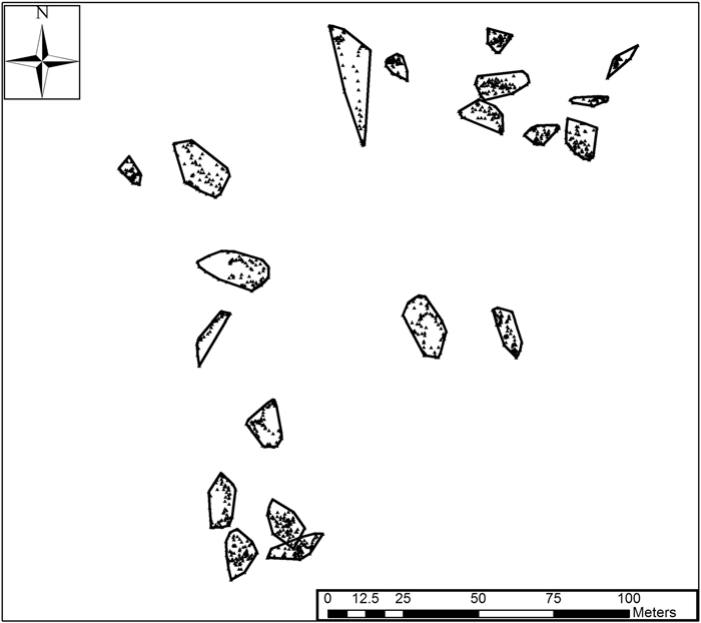
Territories at French Reef. Map of 20 territories (polygons) and GPS tracks (triangles) at French Reef (a protected site).

*Sparisoma aurofrenatum* at protected sites had smaller territories (91.7 ± 5.8 m^2^, mean ± SE, n = 77) relative to those at unprotected sites (131.45 ± 8.9 m^2^, n = 79) (Fig. 2, t = -3.77, df = 133.24, p < 0.001). At all sites, territories generally did not overlap (see Fig. 3 for an example). Predator biomass was low overall, but marginally higher inside protected sites (3.03 g m^-2^, n = 4) relative to unprotected sites (0.66 g m^-2^, n = 4) (t = 2.03, df = 4.57, p = 0.10). Competitor biomass (i.e., *Sparisoma spp*.) was similar inside protected sites (16.4 ± 5.4 g m^-2^, n = 4) and unprotected sites (13.4 ± 3.3 g m^-2^, n = 4) (t = 0.47, df = 4.96, p-value = 0.66).

**Fig 4:**
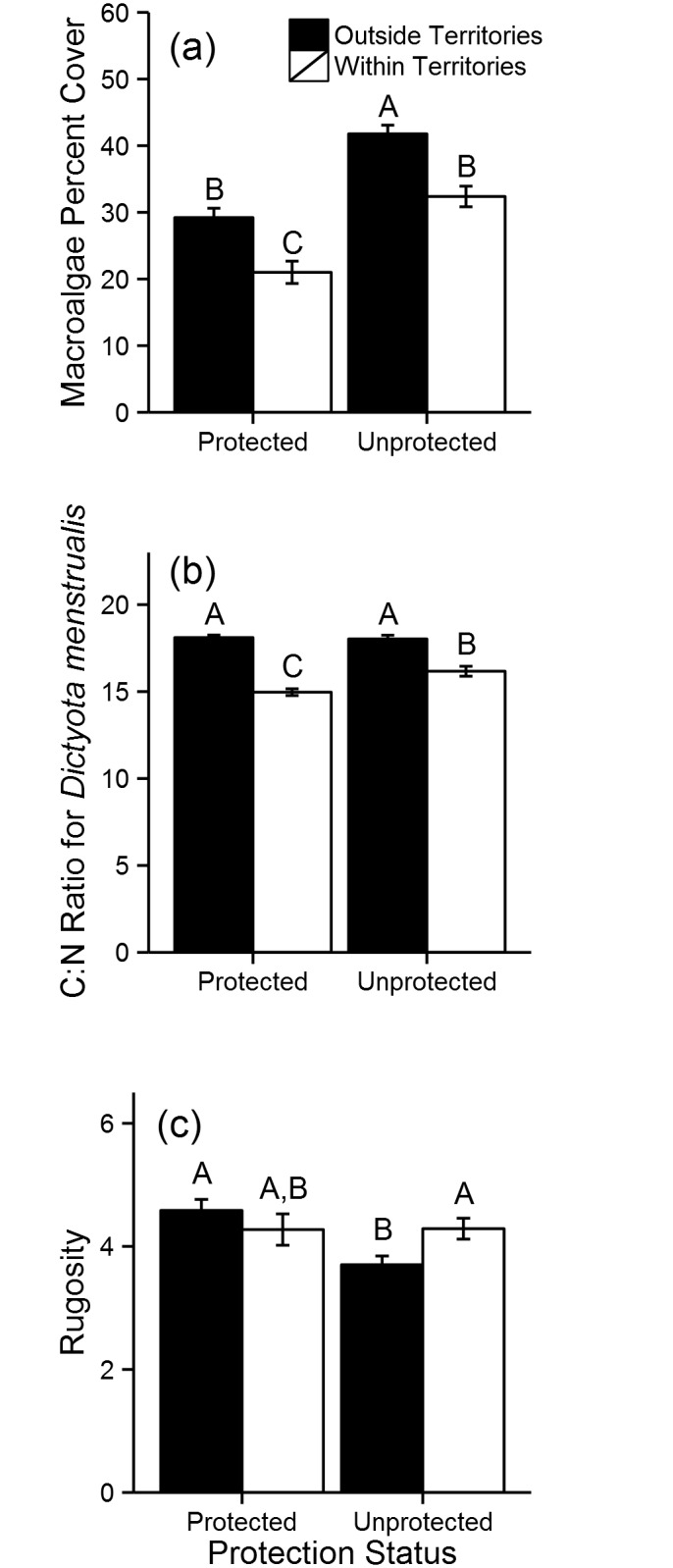
Quality metrics inside and outside of territories. Mean (± SE) (a) Macroalgae cover, (b) C:N ratios of *Dictyota menstrualis*, and (c) LiDAR-derived rugosity outside (n = 80) and inside (n = 77) territories of protected sites and outside (n = 80) and inside (n = 79) territories of unprotected sites. Letters above bars represent differences among groups based on TukeyHSD post-hoc analysis.

Metrics of territory quality (i.e., macroalgal cover, C:N of *D. menstrualis*, and rugosity) varied with territory status (i.e., territory vs. reference) and protection status. Territories had less macroalgae relative to reference areas and, overall, protected sites had less macroalgal cover relative to unprotected sites (Table 1a, Fig. 4a). Pairwise comparisons showed that territories in protected sites had less macroalgal cover relative to territories in unprotected sites. *Dictyota menstrualis* was most nutritious (lower C:N ratios) inside parrotfish territories and particularly inside territories of protected areas (Table 1b, Fig. 4b). Territories in protected and unprotected areas were similar in terms of structural complexity. In unprotected sites territories tended to be more structurally complex relative to reference areas, whereas complexity was similar between territories and reference areas in protected sites (Table 1c, Fig. 4c).

**Table 1:** Results from two-way ANOVAs for differences in (a) macroalgal cover, (b) C:N ratios for *Dictyota menstrualis* and (c) rugosity with protection status (inside and outside of protected areas) and territory status (within and outside of territories).

Response	Factor	F	p
(a) Macroalgae Cover	**Protection Status**	**60.54**	**0.001**
	**Territory Status**	**32.91**	**0.001**
	Protection Status x Territory Status	0.11	0.92
(b) C:N of *D. menstrualis*	**Protection Status**	**5.38**	**0.02**
	**Territory Status**	**123.46**	**0.001**
	**Protection Status x Territory Status**	**8.03**	**0.01**
(c) Rugosity	**Protection Status**	**4.46**	**0.01**
	Territory Status	0.78	0.38
	**Protection Status x Territory Status**	**5.13**	**0.02**

Bold entry indicates significance at the α = 0.05 level.

**Fig 5:**
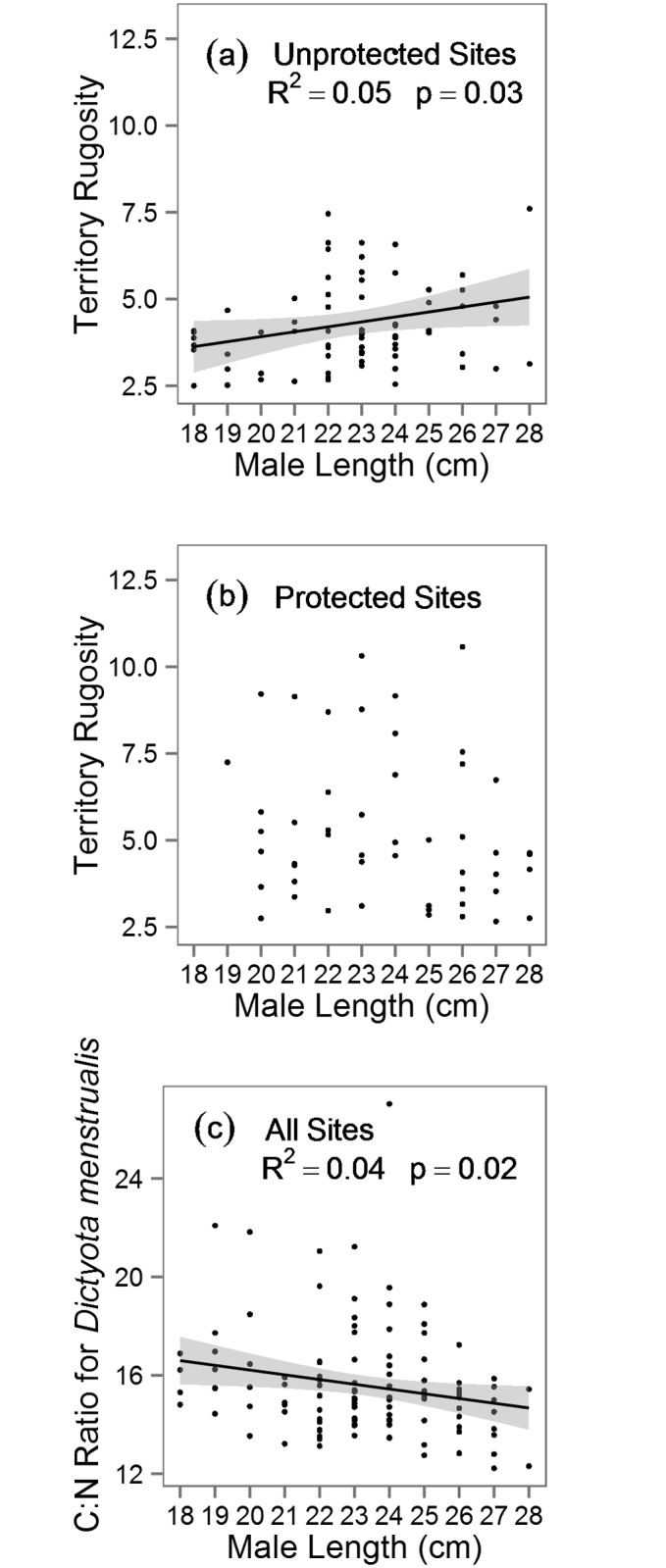
Male size and territory complexity. Male length and territory rugosity in (a) unprotected and (b) protected reefs. In panel (a), solid line represents fitted regression, shaded area represents 95% CI and points represent focal males observed.

We did not find evidence that larger males occupied larger territories or that their territories had more algal resources in either protected or unprotected sites (S2 Table). However larger males had more rugose territories, but only in unprotected areas where this relationship was relatively weak but significant (R^2^ = 0.05, p = 0.03, β = 0.41, SE = 0.18, Fig. 5a&b). Additionally, larger males controlled higher quality territories (lower C:N of *D. menstrualis*), but this relationship was also relatively weak (R^2^ = 0.04, p = 0.02, β = -0.25, SE = 0.09, Fig. 5c). We did not find evidence for any effect of predator biomass on territory size or quality, but competitor biomass was significantly positively associated with territory rugosity (S3 Table).

Territory quality and male size influenced the number and size of females within territories. Overall, males (t = 3.01, df = 148.90, p = 0.003) and harem females (t = 2.48, df = 125.90, p = 0.01) were larger in protected sites, while harem size was similar in both protected and unprotected sites (t = 0.58, df = 151.2, p = 0.56; see S4 Table for summary statistics of male, female and harem sizes between protected and unprotected sites). However, larger harems were associated with larger territories, but unrelated to male length, territory rugosity, algal quality or algal percent cover (Full model: F_5,95_ = 1.48, R^2^ = 0.02, p = 0.20, Table 2a). Larger females were associated with larger males and with territories that had greater algal resource quality (lower C:N ratios), but female size was unrelated to territory area, rugosity or algal percent cover (Full model: F_5,92_ = 10.39, R^2^ = 0.33, p < 0.001, Table 2b).

**Table 2:** Results from multiple regression models for relationships between (a) harem size and (b) average female size and territory quality metrics.

Response	Territory Parameter	β	SE	p
(a) Harem Size	**Area**	**0.96**	**0.47**	**0.05**
	Rugosity	-0.12	0.08	0.14
	C:N *Dictyota menstrualis*	0.03	0.07	0.63
	Macroalgae Percent Cover	0.004	0.05	0.68
	Male Length	0.03	0.05	0.63
(b) Average Female Size	Area	-0.001	0.002	0.40
	Rugosity	-0.11	0.07	0.11
	**C:N *Dictyota menstrualis***	**-0.12**	**0.06**	**0.05**
	Macroalgae Percent Cover	0.009	0.007	0.22
	**Male Length**	**0.25**	**0.04**	**<0.001**

Bold entry indicates significance at the α = 0.05 level.

**Fig 6:**
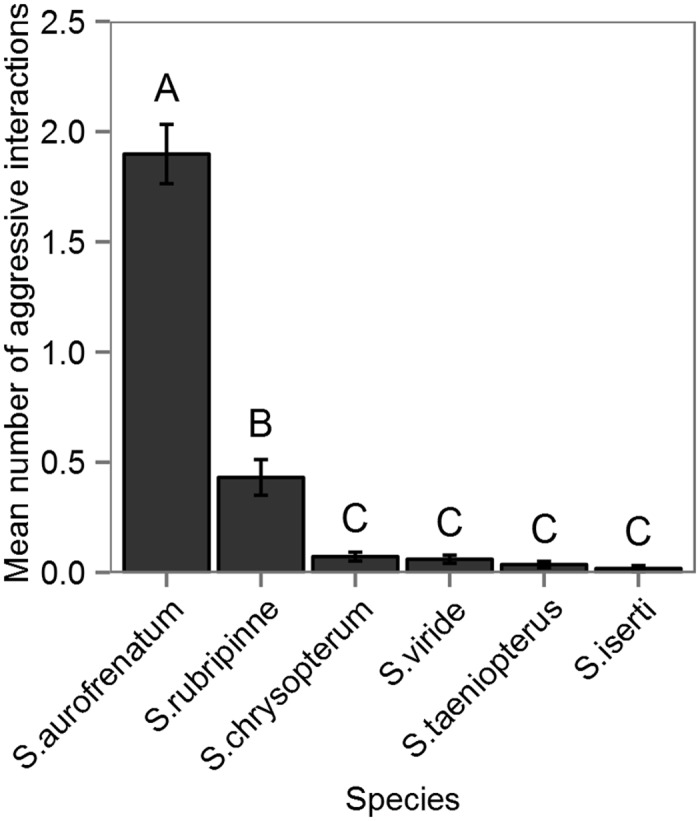
Aggressive interactions with other parrotfishes. Mean (±SE) number of aggressive interactions by focal male with species of parrotfish. Letters above bars represent differences among species based on TukeyHSD post-hoc analysis.

Of the aggressive interactions that focal males exhibited, most involved, rapid chases (52%, 0.13 ± 0.009; % of interactions, mean number per minute ± SE) and fin-flares (38%, 0.09 ± 0.006), while fewer involved parallel swimming (5%, 0.01 ± 0.004) and jaw-fighting (0.5%, 0.005 ± 0.005). All such interactions occurred either within or along the borders of territories. The majority of aggressive interactions initiated by focal males were directed towards conspecifics (~77%). The remaining interactions were with heterospecifics including: *S. rubripinne*, *S. chrysopterum*, *S. viride*, *S. taeniopterus*, and *Scarus iserti* in order of decreasing frequency (Fig. 6). There were significantly more aggressive interactions directed towards conspecifics than other parrotfish species (ANOVA: F_9,996_ = 126.9, p < 0.001). Additionally, based on pairwise comparisons, there were more aggressive interactions directed towards *S. rubripinne* relative to other heterospecifics.

We found that the probability of engaging in aggressive interactions with other parrotfish was positively associated with harem size as we expected (β = 0.45 ± 0.20, p = 0.03). However, contrary to our expectations, the probability of engaging in in aggressive interactions was also positively associated with C:N of *D. menstrualis* (β = 0.25 ± 0.13, p = 0.05) (suggesting territories with lower quality algae were more aggressively defended), and unrelated to average female size or other territory quality metrics (i.e., territory size, rugosity, macroalgal cover) (S5a Table). A Hosmer-Lemeshow test for goodness of fit showed a good fit for the logistic regression model (χ^2^ = 157.42, p = 0.36).

All spawning we observed took place within the focal male’s territory and occurred between the focal male and a female from his harem between 13:00–16:00 h. We observed a total of 32 spawning events by 14 males. Thirty of these spawning events (by 12 males) were observed inside protected sites. Overall, there was a greater proportion of males spawning in protected sites relative to unprotected sites (df = 1, χ^2^ = 6.93, p = 0.008). Spawning males inside protected sites had approximately twice as many spawning episodes relative to those in unprotected sites. We found that the probability of spawning decreased with territory size (β = -0.03, SE = 0.01, p = 0.05), but was unrelated to other territory quality metrics or reef protection status (S5b Table). A Hosmer-Lemeshow test for goodness of fit showed a good fit for the logistic regression model (χ^2^ = 13.05, p = 0.11).

## Discussion

Our work elucidated multiple factors that influence territorial and reproductive behaviors for the parrotfish *S. aurofrenatum* on reefs in the Florida Keys. Reef protection status appeared to underlie some of the differences in quality metrics between territory and reference areas. For instance, there was a difference in algal nutritional quality between territories and surrounding areas (with territories having higher algal nitrogen content) and this difference was greater in protected sites. Reef protection status also influenced territory size, with territories inside protected areas being approximately 25% smaller than those inside unprotected areas. Despite having smaller territories, males inside protected sites and his harem of females tended to be larger, potentially due to greater resource quality in protected territories. Although our data were consistent with the idea that increased predation risk decreased territory size, given smaller territories inside of protected areas, there was no direct correlation between predator biomass and smaller territory size. Our data suggest that it is beneficial for males to maintain large territories with high nutritional quality because they tended to support a greater number of large females. Furthermore, there may be a potential trade-off between territory size and quality as territories in protected sites were smaller but they had algae with higher nutritional quality. However, contrary to our expectations, we observed more spawning activity inside protected areas and in smaller territories. Overall, our work indicates that multiple metrics of both territory quality and male characteristics impact patterns in territoriality and spawning, which may have indirect consequences on the reproductive potential of male territory holders.

Mean territory sizes of *S. aurofrenatum* (112 m^2^), were in the range of those recorded in Belize (82 and 319 m^2^) [19], Puerto Rico (88 m^2^) [42], Barbados (142–215 m^2^) [65], and other areas in Florida (240 m^2^) [43]. Variation in territory sizes measured among these studies may be the result of differences in sampling methodology and/or the type of habitats sampled. Those studies done in Florida and Puerto Rico delineated territories based on locations of aggressive interactions. This method may be less accurate because territories can be maintained through mutual avoidance with or without aggression [66]. Additionally, some of the sites used in the studies from Barbados and Florida were primarily patch reefs, which are likely different in resource distribution relative to contiguous reef structures. Prior studies have also relied on dropping physical markers to delineate territories, which may fail to capture the full extent of the territory and potentially have unintended effects on focal fish behavior. Our methodology using handheld GPSs attached to a float allowed us to more accurately estimate the full extent of male movements with minimal diver interference.

Protection from fishing, which likely increases the abundance of predators of *S. aurofrenatum*, could indirectly affect their territory size. Long-term monitoring of protected areas in the FKNMS has shown increases in absolute and relative predator abundances after reserve implementation as compared to reference areas [36]. The fear of predation could decrease the area over which individuals venture as herbivorous fishes may reduce their excursion area (i.e., the distance or area that individuals move over a given time period) in the presence of increased predation risk [67]. Unlike extensive surveys within the FKNMS [36], we only found marginally greater predator biomass at protected sites compared to unprotected sites. The substantial variation in predator abundances across protected sites, relatively low sample size, or imperfect predator detection [68] may have precluded us from demonstrating a stronger relationship. Territories in protected areas were smaller, consistent with the hypothesis that increased predation risk results in an increased cost of holding larger territories. However, despite being supportive of our hypothesis, predator biomass did not directly relate to territory size or other territory quality metrics. As a consequence of having smaller territories, males likely have a more constrained foraging area and appear to compensate for smaller territories by choosing territories with greater food quality (i.e., lower C:N ratios).

A trade-off between territory size and quality may influence the number and size of harem females. Large territories, which require more surveillance over wide areas, may put *S. aurofrenatum* at greater risk of predation, are likely more energetically expensive to defend, and may be more susceptible to incursions from competitors. The benefit of a large territory is in the greater foraging area it provides and in the greater number of harem females it can support. By choosing areas of the reef with higher algal quality, males may be able to defend smaller more limited foraging areas while still meeting their metabolic needs. Further, despite having fewer females, smaller territories with higher algal quality appear to support larger females with more spawning events. Thus *S. aurofrenatum* appears to make a trade-off between large territories with more feeding opportunities/abundant females and smaller territories with higher algal quality/larger females. These patterns are consistent with our prediction that reef protection, and the subsequent increases in predator biomass [36], would lead to smaller territories even though we only show a marginally significant increase in predator biomass between protected and unprotected areas. Most studies investigating habitat use decisions based on a trade-off between food and safety have focused on either food quantity [69,70] or food quality [71–73]. Our work suggests that the resolution of this trade-off may be more dynamic, with each factor being valued differently depending on context [74].

Despite the clear effect of protection on territory size, we cannot discount the alternative bottom-up explanation that higher algal quality inside territories of protected sites supported smaller territories. It may be more beneficial for males to defend a smaller area, even though there may be fewer potential feeding opportunities, because of the energetic costs to territory defense. Males in protected areas may be better able to realize this advantage because smaller territories tended to also have greater algal nutritional quality. Therefore, smaller territories, which require less defense, may be all that is needed for these males to meet their metabolic needs. Less time spent defending a territory could provide males with more time for other activities such as spawning, which we observed more frequently inside protected areas. However, males in unprotected areas also defended territories with significantly higher algal quality than in surrounding areas. Algal quality within territories in unprotected areas was significantly less than within territories in protected areas. But this difference was very small as compared to the large difference in algal quality in territory vs. non-territory areas in both protected and unprotected areas. It is unclear if the very small differences in algal quality within territories between protected and unprotected areas could explain the large differences in territory size. Further, there need not be one mutually exclusive explanation for the smaller territories in protected areas as both top-down (risk of predation) and bottom-up (algal quality) forces likely shape territory size. Thus, while our data clearly show smaller territories in protected zones, further work will be needed to determine the direction of causation for this relationship.

Structural complexity may also be an important determinant of parrotfish territories. This was especially the case in unprotected areas where structurally complex habitat was more limited and males targeted high complexity areas with larger males securing the most sought-after and complex territories. Large males are likely superior competitors and potentially at less risk of predation, making them better able to secure quality territories [5]. These more complex areas may make better territories because more complexity typically attracts more fishes [40], and fish aggregations may have an indirect positive effect on food resources by increasing nitrogen availability from fish excretion for macroalgae within those areas. In the Florida Keys fish excretion can supply up to 25 times more nitrogen to forereefs than all other biotic and abiotic sources combined [49]. Further, higher biomass of fishes was associated with decreased algal C:N (higher algal nutritional quality) at the reef-wide scale [49]. Thus, aggregations of fishes associating with highly complex regions inside territories, may have increased the nitrogen supply available to the benthos leading to higher algal nutritional quality inside territories. This would provide an added benefit for *S. aurofrenatum* in choosing high complexity regions to establish their territories. Thus, more complex areas are likely more desirable as a feeding and breeding habitat for *S. aurofrenatum*, as has been demonstrated in other reef fish [75]. As coral cover declines [76] and reef complexity is lost [77], our data suggests that there also may be an associated loss of key habitat types for territorial species.

Contrary to what we expected, the probability of males engaging in aggressive interactions was not positively associated with metrics of territory quality. This may be due to the overall low probability of aggressive interactions or imperfect detection of aggression by observers. Territories can be maintained without overt aggression, with males avoiding potentially injurious interactions through mutual avoidance [66]. It is possible that observers may have overlooked such subtle avoidance behaviors while watching for more overt aggression, resulting in an under-sampling of territorial interactions.

Our data also suggest that protection status may influence reproductive behaviors. Spawning was almost exclusively observed in protected sites, even though we consistently made observations covering the same time periods at all sites (10:00–16:00 h). In particular, the two protected sites with the highest predator biomass (French Reef and Molasses Reef) also had the greatest proportion of males spawning (25% and 11%, respectively) relative to other sites. This was contrary to what we expected, as others have shown that the increased risk of predation suppresses courtship activity in fishes (e.g., [28,31,32,78]). It is possible that *S. aurofrenatum* in protected sites spawn during mid-day to avoid crepuscular predators, as opposed to dusk when spawning activity generally peaks [21,42]. This may explain why we did not observe spawning in unprotected sites during our mid-day surveys, but without dusk observations we can only speculate about peaks in spawning at unprotected sites.

However, there are multiple, likely interacting, characteristics of protected sites that could also explain the increased likelihood of spawning. Inside protected areas there was greater structural complexity, territories were smaller and had food resources with greater nutritional quality relative to unprotected sites. For many coral reef fish, sites with high substratum rugosity are a preferred microhabitat for spawning behavior [75], although we could not detect a direct relationship between structural complexity and the frequency of spawning. Furthermore, males may be more likely to encounter and thus spawn with harem females in smaller territories, which we found in protected areas. Also, higher food quality, which we also found in territories in protected areas, may allow males to meet the energetically expensive demands of spawning. Additionally, there are other factors occurring at different spatial and temporal scales that we did not measure that can influence spawning activity of coral reef fish including reef size, the availability of suitable spawning sites, and the potential for successful transport of gametes [75,79,80]. These factors likely varied among study sites, individual territories, and sampling days, making it challenging to identify all of the potential drivers of spawning activity.

Overall, our data support the idea that territoriality in *S. aurofrenatum* is at least partially linked to food resources, which likely has consequences for reproduction. We show that males choose territories based on food resources because: (1) algal nutritional quality was greater inside territories, (2) aggression was primarily targeted towards individuals with the greatest resource overlap (i.e., conspecifics and congeners) [23,41], and (3) territories with the highest algal nutritional quality were defended by the largest, competitively superior males. The purpose for gaining exclusive access to food resources seemed to be to enhance reproductive potential because: (1) large territories were associated with larger harems and (2) high quality algae attracted larger females. It has been demonstrated in multiple other species (i.e., great tits [3], field sparrows [12], redwinged blackbirds [10] and European roe deer [11]) that territory size and quality influences breeding success, however data on reef fish is limited [81–83]. Importantly, we show that multiple, interactive factors associated with protection status including, resource quality, and reef structural complexity as well as male characteristics, shape territoriality in *S. aurofrenatum*.

Along multiple reefs in the Florida Keys, variability in territory size and quality of *S. aurofrenatum* between protected and unprotected sites, suggests a trade-off between the costs and benefits maintaining exclusive access to feeding and breeding grounds. Inside protected sites where predators are often more abundant [36] and reefs are more structurally complex, territories are smaller and have higher resource quality. Whereas, inside unprotected sites that often have fewer predators and less complex reef structure, territories are larger but have lower resource quality and are equally as rugose as territories in protected sites. These differences suggest that greater resource quality offsets constraints in territory size which could be driven by increased predation risk. Although we did not find direct relationships between predator biomass and territory metrics, we demonstrate patterns associated with reef protection status that support this hypothesis. In recent decades the decline in coral abundance on coral reefs due to multiple global and local stressors [84] have been associated with dramatic declines in reef structural complexity [77] and predator biomass [85]. The indirect effects of coral reef declines, particularly on social and reproductive interactions of reef associated species, is yet unclear. However, our data suggest that the reduction in rugosity resulting from coral loss and the changing abundances of predators from overfishing could alter the territory dynamics of important herbivorous fishes.

## Supporting Information

S1 Fig Observation time and territory size. Territory areas (m^2^) calculated every five minutes over the course of thirty minutes for four individual *S. aurofrenatum*.
(TIFF)

S1 Table Summary of study sites. Study sites with GPS coordinates, protection status (Protected (P) or Not Protected (NP), macroalgae % cover, turf/algae/sediment (TAS) % cover, coral % cover, competitor and predator biomass and LiDAR-derived rugosity.
(DOCX)

S2 Table Summary of ANCOVA models for the influence of male length and protection status on territory quality variables. Bold entry indicates significance at the α = 0.05 level.
(DOCX)

S3 Table Summary of mixed-effects models for territory quality variables and site level predictors of predator and competitor biomass. Bold entry indicates significance at the α = 0.05 level.
(DOCX)

S4 Table Means ± SE of male size, female size and harem size in protected and unprotected sites. Bold entry indicates significant differences with status at the α = 0.05 level.
(DOCX)

S5 Table Summary of multiple logistic regression model for the probability of (a) engaging in aggressive interactions and (b) spawning, with territory quality variables, harem size, female size and reef protection status. Bold entry indicates significance at the α = 0.05 level.
(DOCX)
