# Supplementary figures and images for: Predation Risk, Resource Quality, and Reef Structural Complexity Shape Territoriality in a Coral Reef Herbivore
Source: PLoS One. 2015 Feb 25;10(2):e0118764. doi: 10.1371/journal.pone.0118764 (PMC4340949; doi:10.1371/journal.pone.0118764)

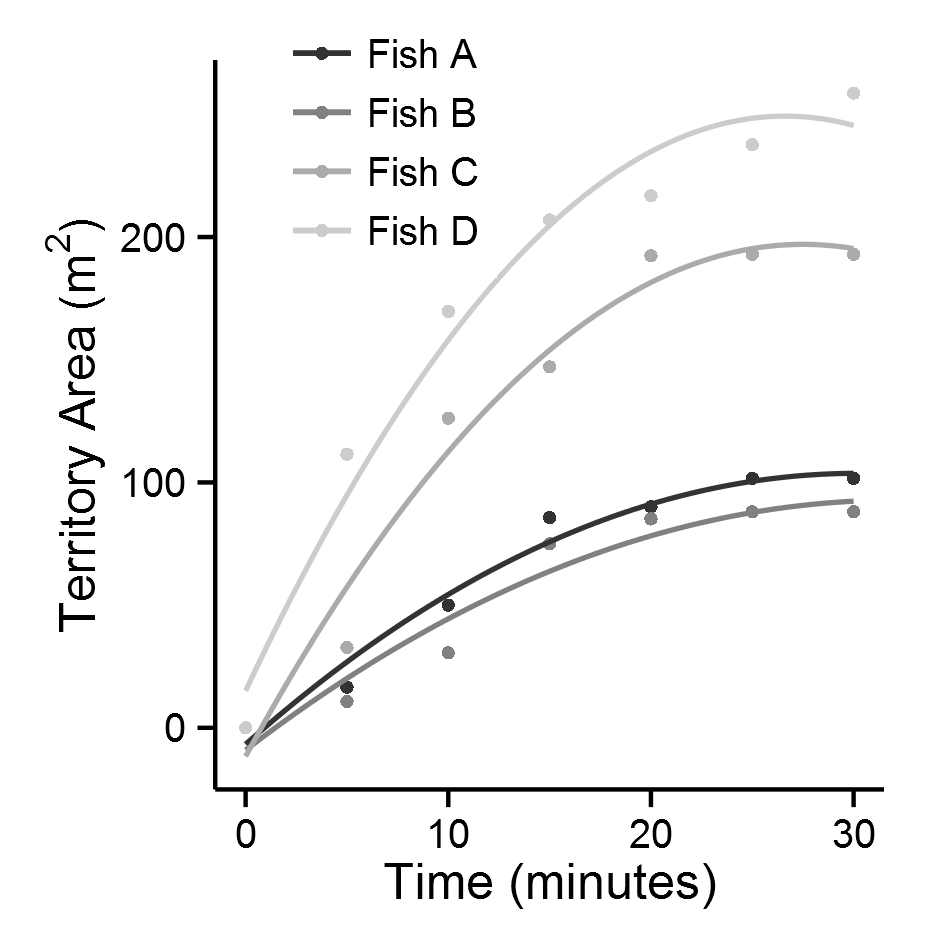

Supplement: S1 Fig — Territory areas (m2) calculated every five minutes over the course of thirty minutes for four individual S. aurofrenatum. (TIFF) [file pone.0118764.s001.tiff]
