# Supplementary material for: Predation Risk, Resource Quality, and Reef Structural Complexity Shape Territoriality in a Coral Reef Herbivore
Source: PLoS One. 2015 Feb 25;10(2):e0118764. doi: 10.1371/journal.pone.0118764 (PMC4340949; doi:10.1371/journal.pone.0118764)
Supplement: S1 Table — Study sites with GPS coordinates, protection status (Protected (P) or Not Protected (NP), macroalgae % cover, turf/algae/sediment (TAS) % cover, coral % cover, competitor and predator biomass and LiDAR-derived rugosity. (DOCX) [file pone.0118764.s002.docx]

**Table S1** – **Study sites with GPS coordinates, protection status (Protected (P) or Not Protected (NP), macroalgae % cover, turf/algae/sediment (TAS) % cover, coral % cover, competitor and predator biomass and LiDAR-derived rugosity.**

| **Site** | **Lat.** | **Long.** | **Status** | **Macro-algae** (% cover) | **TAS (% cover)** | **Coral** (% cover) | **Competitor Biomass** (g m^-2^) | **Predator Biomass** (g m^-2^) | **LiDAR-derived Rugosity** |
| --- | --- | --- | --- | --- | --- | --- | --- | --- | --- |
| Conch | 24.96 | -80.46 | P | 40.8 | 30.8 | 0.33 | 7.3 | 0 | 3.54 |
| French | 25.04 | -80.36 | P | 35.4 | 18.4 | 3.27 | 16.3 | 3.53 | 3.66 |
| S. Carysfort | 25.21 | -80.22 | P | 31.9 | 31.8 | 2.63 | 31.7 | 3.41 | 4.52 |
| Molasses | 25.01 | -80.38 | P | 8.8 | 30.0 | 1.30 | 10.4 | 5.06 | 3.52 |
| Pickles | 24.99 | -80.41 | NP | 25.2 | 50.9 | 1.33 | 21.3 | 0.34 | 3.42 |
| Pinnacles | 24.99 | -80.41 | NP | 39.3 | 44.4 | 1.00 | 13.8 | 0 | 3.15 |
| Maitland | 25.19 | -80.23 | NP | 55.3 | 24.8 | 1.48 | 5.1 | 0 | 3.96 |
| Snapper Ledge | 24.99 | -80.42 | NP | 47.3 | 28.4 | 0.95 | 13.6 | 2.29 | 2.84 |
