# Supplementary material for: Predation Risk, Resource Quality, and Reef Structural Complexity Shape Territoriality in a Coral Reef Herbivore
Source: PLoS One. 2015 Feb 25;10(2):e0118764. doi: 10.1371/journal.pone.0118764 (PMC4340949; doi:10.1371/journal.pone.0118764)
Supplement: S2 Table — Bold entry indicates significance at the α = 0.05 level. (DOCX) [file pone.0118764.s003.docx]

**Table S2** – **Summary of ANCOVA models for the influence of male length and protection status on territory quality variables.**

| **Territory Quality Variable** | **Factor** | **Estimate** | **SE** | **P** |
| --- | --- | --- | --- | --- |
| Area | Male Length | 3.65 | 2.86 | 0.20 |
|  | **Protection Status** | **166.62** | **98.84** | **0.05** |
|  | Male Length X Protection Status | -5.29 | 4.23 | 0.21 |
| Rugosity | Male Length | -0.11 | 0.08 | 0.18 |
|  | **Protection Status** | **-6.17** | **2.81** | **0.03** |
|  | **Male Length X Protection Status** | **0.26** | **0.12** | **0.03** |
| Macroalgae Cover | Male Length | 0.30 | 0.67 | 0.66 |
|  | Protection Status | 24.94 | 23.22 | 0.29 |
|  | Male Length X Protection Status | -0.58 | 0.99 | 0.56 |
| C:N *D. menstrualis* | **Male Length** | **-0.22** | **0.11** | **0.05** |
|  | Protection Status | -2.61 | 3.90 | 0.50 |
|  | Male Length X Protection Status | 0.15 | 0.17 | 0.35 |
