# Supplementary material for: Predation Risk, Resource Quality, and Reef Structural Complexity Shape Territoriality in a Coral Reef Herbivore
Source: PLoS One. 2015 Feb 25;10(2):e0118764. doi: 10.1371/journal.pone.0118764 (PMC4340949; doi:10.1371/journal.pone.0118764)
Supplement: S3 Table — Bold entry indicates significance at the α = 0.05 level. (DOCX) [file pone.0118764.s004.docx]

**Table S3** – **Summary of mixed-effects models for territory quality variables and site level predictors of predator and competitor biomass.**

| **Territory Quality Variable** | **Factor** | **Estimate** | **SE** | **P** |
| --- | --- | --- | --- | --- |
| Area | Predator Biomass | 1.03 | 3.74 | 0.79 |
|  | Competitor Biomass | -0.71 | 0.92 | 0.48 |
| Rugosity | Predator Biomass | -0.02 | 0.18 | 0.94 |
|  | **Competitor Biomass** | **0.16** | **0.05** | **0.04** |
| Macroalgae Cover | Predator Biomass | -1.42 | 2.11 | 0.54 |
|  | Competitor Biomass | 0.86 | 0.63 | 0.24 |
| C:N *D. menstrualis* | Predator Biomass | -0.18 | 0.17 | 0.37 |
|  | Competitor Biomass | -0.04 | 0.05 | 0.41 |
