# Supplementary material for: Predation Risk, Resource Quality, and Reef Structural Complexity Shape Territoriality in a Coral Reef Herbivore
Source: PLoS One. 2015 Feb 25;10(2):e0118764. doi: 10.1371/journal.pone.0118764 (PMC4340949; doi:10.1371/journal.pone.0118764)
Supplement: S4 Table — Bold entry indicates significant differences with status at the α = 0.05 level. (DOCX) [file pone.0118764.s005.docx]

**Table S4 – Mean ± SE of male size, female size and harem size among protected and unprotected sites.**

|  | Protected | Unprotected |
| --- | --- | --- |
| Male Size (cm) | **23.92 ± 0.31** | **22.66 ± 0.28** |
| Female Size (cm) | **15.59 ± 0.18** | **15.06 ± 0.12** |
| Harem Size (# indiv) | 4.22 ± 0.17 | 4.09 ± 0.15 |
