# Supplementary material for: Predation Risk, Resource Quality, and Reef Structural Complexity Shape Territoriality in a Coral Reef Herbivore
Source: PLoS One. 2015 Feb 25;10(2):e0118764. doi: 10.1371/journal.pone.0118764 (PMC4340949; doi:10.1371/journal.pone.0118764)
Supplement: S5 Table — Bold entry indicates significance at the α = 0.05 level. (DOCX) [file pone.0118764.s006.docx]

**Table S5** – **Summary of multiple logistic regression model for the probability of (a) engaging in aggressive interactions and (b) spawning, with territory quality variables, harem size, female size and reef protection status.**

| **Response** | **Factor** | **Estimate** | **SE** | **P** |
| --- | --- | --- | --- | --- |
| (a) Aggressive Interactions | Area | 0.00 | 0.00 | 0.21 |
|  | Rugosity | -0.21 | 0.16 | 0.19 |
|  | Macroalgal Cover | 0.01 | 0.02 | 0.51 |
|  | **C:N *Dictyota menstrualis*** | **0.25** | **0.13** | **0.05** |
|  | **Harem Size** | **0.45** | **0.20** | **0.03** |
|  | Average Female Size | 0.11 | 0.19 | 0.55 |
|  | Reef Protection Status | 0.25 | 0.49 | 0.62 |
| (b) Spawning | **Area** | **-0.03** | **0.01** | **0.02** |
|  | Rugosity | 0.03 | 0.25 | 0.89 |
|  | Macroalgal Cover | 0.01 | 0.03 | 0.84 |
|  | C:N *Dictyota menstrualis* | 0.08 | 0.25 | 0.74 |
|  | Harem Size | 0.39 | 0.39 | 0.31 |
|  | Average Female Size | -0.15 | 0.32 | 0.63 |
|  | Reef Protection Status | -19.0 | 2272.6 | 0.99 |
